# A K_ATP_ Channel-Dependent Pathway within α Cells Regulates Glucagon Release from Both Rodent and Human Islets of Langerhans 

**DOI:** 10.1371/journal.pbio.0050143

**Published:** 2007-05-15

**Authors:** Patrick E MacDonald, Yang Zhang De Marinis, Reshma Ramracheya, Albert Salehi, Xiaosong Ma, Paul R. V Johnson, Roger Cox, Lena Eliasson, Patrik Rorsman

**Affiliations:** 1 Oxford Centre for Diabetes, Endocrinology and Metabolism, University of Oxford, Oxford, United Kingdom; 2 Department of Pharmacology, Faculty of Medicine and Dentistry, University of Alberta, Edmonton, Alberta, Canada; 3 Lund University Diabetes Centre, Clinical Research Center, Malmö, Sweden; 4 Medical Research Council Mammalian Genetics Unit, Harwell, United Kingdom; University of Cambridge, United Kingdom

## Abstract

Glucagon, secreted from pancreatic islet α cells, stimulates gluconeogenesis and liver glycogen breakdown. The mechanism regulating glucagon release is debated, and variously attributed to neuronal control, paracrine control by neighbouring β cells, or to an intrinsic glucose sensing by the α cells themselves. We examined hormone secretion and Ca^2+^ responses of α and β cells within intact rodent and human islets. Glucose-dependent suppression of glucagon release persisted when paracrine GABA or Zn^2+^ signalling was blocked, but was reversed by low concentrations (1–20 μM) of the ATP-sensitive K^+^ (K_ATP_) channel opener diazoxide, which had no effect on insulin release or β cell responses. This effect was prevented by the K_ATP_ channel blocker tolbutamide (100 μM). Higher diazoxide concentrations (≥30 μM) decreased glucagon and insulin secretion, and α- and β-cell Ca^2+^ responses, in parallel. In the absence of glucose, tolbutamide at low concentrations (<1 μM) stimulated glucagon secretion, whereas high concentrations (>10 μM) were inhibitory. In the presence of a maximally inhibitory concentration of tolbutamide (0.5 mM), glucose had no additional suppressive effect. Downstream of the K_ATP_ channel, inhibition of voltage-gated Na^+^ (TTX) and N-type Ca^2+^ channels (ω-conotoxin), but not L-type Ca^2+^ channels (nifedipine), prevented glucagon secretion. Both the N-type Ca^2+^ channels and α-cell exocytosis were inactivated at depolarised membrane potentials. Rodent and human glucagon secretion is regulated by an α-cell K_ATP_ channel-dependent mechanism. We propose that elevated glucose reduces electrical activity and exocytosis via depolarisation-induced inactivation of ion channels involved in action potential firing and secretion.

## Author Summary

Glucagon is a critical regulator of glucose homeostasis. Its major action is to mobilize glucose from the liver. Glucagon secretion from α cells of the pancreatic islets of Langerhans is suppressed by elevated blood sugar, a response that is often perturbed in diabetes. Much work has focused on the regulation of α-cell glucagon secretion by neuronal factors and by paracrine factors from neighbouring cells, including the important islet hormone insulin. In contrast, we provide evidence in support of a direct effect of glucose on α cells within intact rodent and human islets. Notably, our work implicates an α-cell glucose-sensing pathway similar to that found in insulin-secreting β cells, involving closure of ATP-dependent K^+^ channels in the presence of glucose. Furthermore, we find that membrane depolarisation results in inhibition of Na^+^ and Ca^2+^ channel activity and α-cell exocytosis. Thus, we propose that elevated blood glucose reduces α-cell electrical activity and glucagon secretion by inactivating the ion channels involved in action potential firing and secretion.

## Abstract 3

Elevated glucose levels reduce electrical activity and the release of glucagon via inactivation of ion channels in pancreatic islet cells.

## Introduction

Blood glucose levels are under the control of two hormones released from the pancreatic islets of Langerhans. Islet β cells secrete insulin when glucose is high, decreasing glucose production by the liver and increasing glucose storage in multiple tissues. Regulated insulin secretion is relatively well understood, involving the metabolic stimulation of electrical activity, Ca^2+^ entry, and exocytosis [1]. Islet α cells secrete glucagon in response to *decreased* blood glucose, whereas elevated glucose levels suppress glucagon release. Glucagon is the principal factor stimulating glucose production by the liver. In diabetes, baseline glucagon release is elevated, and glucagon secretion in the low-glucose condition is blunted [2–4]. These effects contribute to chronic hyperglycaemia and to an increased risk for acute hypoglycaemic events.

The mechanism regulating glucagon secretion is poorly understood and remains hotly debated [5]. Glucagon release in rodents may be regulated by paracrine signals, including γ-aminobutyric acid (GABA) [6,7], Zn^2+^ [8], and insulin [9,10]. Conversely, glucose may suppress glucagon secretion through a direct effect on α-cell activity [11–14]. There are also studies suggesting that glucagon secretion is under hypothalamic control [15,16]. Human in vivo studies provide conflicting evidence regarding the control of glucagon secretion by paracrine or intrinsic regulation of α cells [17–30], and very little work has been done to examine this question in isolated human islets.

Islet α cells express ATP-dependent K^+^ (K_ATP_) channels [9,13,14,31,32] that can be closed by ATP [9,31]. Glucose increases intracellular free ATP in α cells [8,10], although reports vary as to the ability of glucose to inhibit α-cell K_ATP_ channels [13,31,33]. Evidence from SUR1^−/−^ mice implicate K_ATP_ channels as regulators of glucagon release [13,34,35]. However, because both α and β cells possess molecularly identical K_ATP_ channels [36,37], it is not clear how K_ATP_-mediated depolarisation would stimulate insulin secretion but suppress glucagon secretion.

The answer may lie in the downstream machinery regulating electrical activity and Ca^2+^ entry. Unlike β cells, α cells possess a large voltage-dependent Na^+^ current that is essential for glucagon release [14,33]. We have previously proposed that the depolarisation-induced inactivation of this channel contributes to the cessation of action potential firing [14]. Additionally, activation of voltage-dependent Ca^2+^ channels (VDCCs) is essential for Ca^2+^ entry and α-cell function [38,39]. Accordingly, the α-cell intracellular Ca^2+^ ([Ca^2+^]_i_) oscillations (reflecting α-cell electrical activity) are suppressed in parallel with glucagon release by glucose [40]. Multiple VDCCs regulate glucagon release [14,41–43], although the N-type channels appear to be particularly important for glucagon release evoked by hypoglycaemia alone [33,44,45], at least in mouse islets. This is in contrast to the β cell in which the L-type VDCC functionally predominates [46].

We have now compared insulin and glucagon release and α- and β-cell Ca^2+^ responses in intact mouse, rat, and human pancreatic islets. We show that glucose retained the ability to suppress glucagon release from isolated islets during blockade of the Zn^2+^ and GABA paracrine pathways, and in the absence of stimulated insulin secretion or β-cell Ca^2+^ responses. Thus we now provide evidence in both rodent and human islets supporting the direct (intrinsic) glucose regulation of glucagon release from pancreatic α cells.

## Results

### Glucose Can Regulate Glucagon Secretion Directly

**Figure 1:**
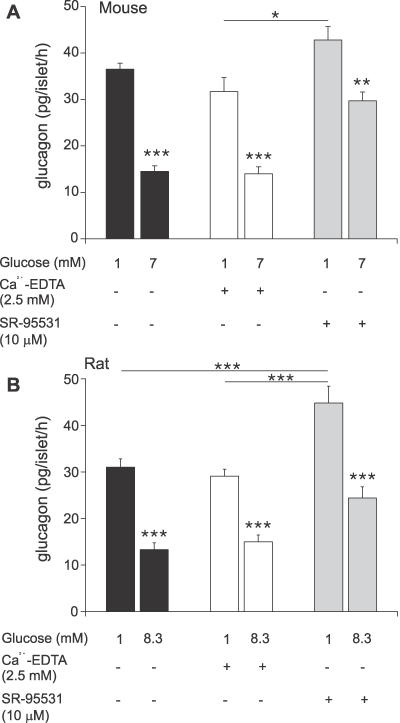
Glucose Suppresses Glucagon Release Independently of Paracrine Signals Mediated by Zn^2+^ or GABA. (A) Glucagon release from isolated mouse islets was suppressed by 60% at 7 mM glucose compared with 1 mM (filled bars). Glucose retained its suppressive effect on glucagon release under conditions of Zn^2+^ chelation (Ca^2+^-EDTA) (open bars) and antagonism of GABA_A_ receptors (SR-95531) (shaded bars). Antagonism of GABA_A_ receptors increased both basal and glucose-suppressed glucagon secretion, suggesting a paracrine role for GABA independent of the glucose effect. (B) As in (A), but using rat islets. *, p < 0.05; **, *p* < 0.01; ***, *p* < 0.001, compared with 1 mM glucose, or as indicated.

To examine a role for GABA and Zn^2+^ as paracrine mediators of glucagon secretion, we examined the ability of the GABA_A_ receptor antagonist SR-95531 and Zn^2+^ chelation with Ca^2+^-EDTA to prevent the glucose-dependent suppression of glucagon release. Glucose, at concentrations (7 and 8.3 mM) just above the threshold for insulin release (see below), suppressed glucagon secretion from isolated mouse (Figure 1A) and rat islets (Figure 1B) by 60% (*n* = 15, *p* < 0.001) and 57% (*n* = 10, *p* < 0.001), respectively. In both mouse and rat islets, the ability of glucose to inhibit glucagon secretion persisted in the presence of Ca^2+^-EDTA (43% and 48%, *n* = 10, *p* < 0.001, respectively) and SR-95531 (31% and 46%, *n* = 8 and 10, *p* < 0.01 and *p* < 0.001, respectively) (Figure 1).

It is worth noting that in the presence of the GABA_A_ antagonist, glucagon secretion was increased under both low- and high-glucose conditions, and furthermore, glucose was approximately 50% less effective in suppressing glucagon release from mouse islets (31% versus 60%, respectively) (Figure 1A). Additionally, somatostatin released from pancreatic δ cells is suggested to be a potential paracrine regulator of glucagon secretion. However, the somatostatin receptor 2 (SSTR-2) antagonist PRL-2903 does not interfere with the ability of glucose (at 3 and 7 mM) to inhibit glucagon secretion from mouse islets [47]. Like the GABA_A_ receptor antagonist, however, PRL-2903 increased glucagon secretion in low-glucose conditions [47]. Thus, although the present data do not entirely rule out these pathways as modulators of glucagon secretion, glucose is clearly able to suppress glucagon secretion independently of these.

### α-Cell K_ATP_ Channels Regulate Glucagon Secretion

**Figure 2:**
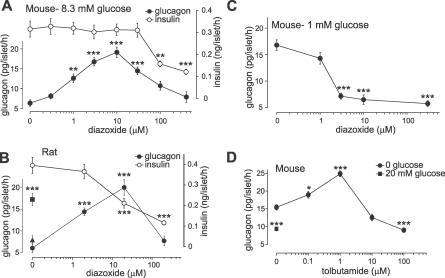
Glucagon Release from Isolated and Intact Mouse Islets Is Regulated by a K_ATP_ Channel-Dependent Pathway (A) Glucagon (filled circles) and insulin (open circles) secretion measured from mouse islets in the presence of 8.3 mM glucose at increasing concentrations of diazoxide. (B) As in (A), but using rat islets. The glucagon responses to 1 mM glucose (filled square) and 100 μM tolbutamide (filled triangle) are indicated. (C) As in (A), but in the presence of 1 mM glucose and only measuring glucagon secretion. (D) As in (A), but examining the effect of tolbutamide in the absence of glucose. Glucagon secretion in response to 20 mM glucose is indicated by the filled square. *, *p* < 0.05; **, *p* < 0.01; ***, *p* < 0.001, compared with zero diazoxide (A–C) or zero tolbutamide (D).

We next examined glucagon and insulin secretion during pharmacological manipulation of K_ATP_ channel activity. Here we have examined the role of K_ATP_ channels in intact islets by applying an indirect, but minimally invasive, technique. It would have been difficult (if not impossible) to study the effects of glucose on α-cell K_ATP_ channel activity using the patch-clamp technique because of the smallness of the α-cell resting conductance (0.15 nS/pF in the absence of glucose, of which two thirds is attributable to K_ATP_ channels) [13]. We have instead used increasing concentrations of diazoxide and tolbutamide to “titrate” the influence of K_ATP_ channel activity on α-cell [Ca^2+^]_i_ and glucagon secretion. Increasing concentrations of the K_ATP_ channel activator diazoxide demonstrated that moderate activation of K_ATP_ channels (0.3–10 μM diazoxide) relieved the suppression of glucagon secretion from both mouse (Figure 2A) and rat (Figure 2B) islets. Stimulation of glucagon release was half-maximal at approximately 1 μM diazoxide, which is well below that required to inhibit insulin release, suggesting that “re-activation” of glucagon release was not secondary to reduced β-cell secretion. Increasing the concentration of diazoxide beyond 10 μM inhibited glucagon secretion in parallel with an inhibition of insulin release from both mouse and rat islets (Figure 2A and 2B). When instead applied in the presence of 1 mM glucose (at which concentration glucagon secretion is stimulated), increasing diazoxide produced a monotonic inhibition of glucagon secretion (Figure 2C) with a half-maximal inhibitory concentration (IC_50_) that is much lower than what is seen under high-glucose conditions (∼2 μM versus ∼50 μM).

**Figure 3:**
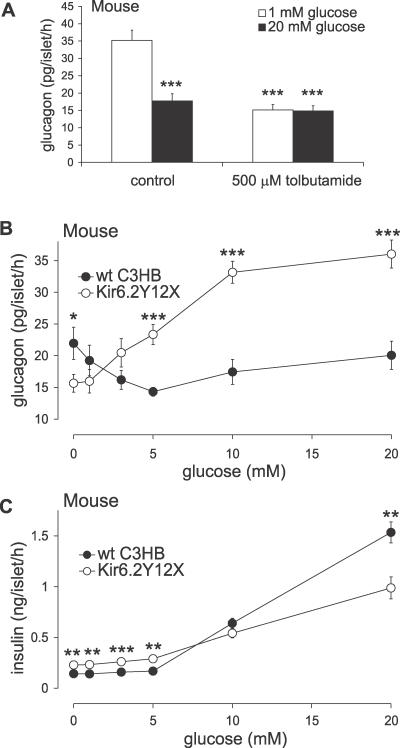
Tolbutamide and Glucose Effects Are Non-Additive and Glucagon Response Is Altered in Kir6.2Y12X Islets That Express a Truncated Kir6.2 Subunit (A) Glucagon release from isolated mouse islets at 1 (open bars) and 20 mM glucose (filled bars) under control conditions and presence of 0.5 mM tolbutamide. (B) A glucose dose-response of glucagon release from control C3HB islets (filled circles) and Kir6.2Y12X islets (open circles). (C) As in (B), but insulin was measured. *, *p* < 0.05; **, *p* < 0.01; ***, *p* < 0.001, compared with control.

In the complete absence of glucose, when K_ATP_ channels are expected to be open, the K_ATP_ channel antagonist tolbutamide also produced a biphasic effect on glucagon release. Augmentation of glucagon secretion was seen at tolbutamide concentrations of up to 1 μM (stimulation being half-maximal at 0.1 μM), whereas inhibition was observed at higher concentrations (Figure 2D). Importantly, in the presence of a maximally inhibitory tolbutamide concentration (0.5 mM), glucose was unable to produce any further inhibition of glucagon release (Figure 3A). These data are inconsistent with the idea that β-cell secretion is the primary inhibitor of α-cell glucagon release. They also suggest that glucagon secretion is maximal within a “window” of intermediate K_ATP_ channel activity.

To further investigate the role of α-cell K_ATP_ channels as regulators of glucagon secretion, we studied islets from Kir6.2Y12X mice, which posses a Tyr12STOP mutation in the *Kcnj11* gene, leading to premature termination of the K_ATP_ channel pore-forming subunit [48]. At low-glucose concentrations, glucagon secretion from the Kir6.2Y12X islets was already suppressed compared with that from wild-type islets (Figure 3B), similar to the effect of glucose stimulation or pharmacological K_ATP_ channel inhibition observed in Figures 2D and 3A. Consistent with a recent report [49], higher glucose levels stimulated glucagon secretion, perhaps due to a direct effect of metabolism on secretion [33]. In wild-type islets, glucagon secretion exhibited a nadir at 5 mM glucose, and glucagon secretion at 20 mM glucose was 40% higher than at 5 mM glucose. Insulin secretion in the Kir6.2Y12X islets was elevated at low-glucose concentrations (Figure 3C), whereas glucose-stimulated insulin secretion was blunted. It is notable that the increase in glucagon release from the Kir6.2Y12X islets was coincident with increased insulin secretion, reinforcing the view that inhibition of glucagon is not mediated by a factor released by the β cells. Furthermore, in control islets, the inhibition of glucagon secretion is maximal at a concentration of glucose (5 mM) that is without stimulatory action on insulin secretion.

**Figure 4:**
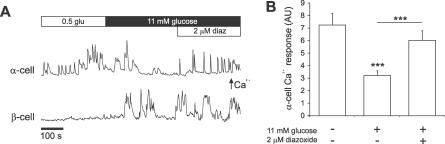
The Intracellular Ca^2+^ Response of Single α Cells within Intact Islets Can Be Re-Activated by Low Concentrations of Diazoxide (A) Representative intracellular Ca^2+^ responses from α and β cells within an intact mouse islet exposed to 0.5 mM glucose, 11 mM glucose, and 11 mM glucose plus 2 μM diazoxide (diaz) as indicated above the traces. (B) The Ca^2+^ response of α cells was suppressed by 11 mM glucose, and could be reactivated with low concentrations of the K_ATP_ channel agonist diazoxide. ***, *p* < 0.001, compared with the low-glucose condition, or as indicated.

We next examined the function of α and β cells in situ by monitoring the [Ca^2+^]_i_ responses of single cells within intact mouse islets. These were functionally identified by their [Ca^2+^]_i_ response to 0.5, 2, and 11 mM glucose [50]. Glucose stimulation suppressed the [Ca^2+^]_i_ response of α cells by 51 ± 3% (*n* = 63 cells in 15 islets, *p* < 0.001) (Figure 4A and 4B). The suppressive effect of glucose was alleviated (to 101 ± 11% of initial values) by application of 2 μM diazoxide (Figure 4A and 4B). This concentration of diazoxide was similar to that which produced maximal stimulation of glucagon secretion and much (∼15-fold) lower than necessary to inhibit insulin secretion (Figure 2B) or β-cell [Ca^2+^]_i_ responses to glucose (Figure 4A).

### K_ATP_ Channels Regulate Human α-Cell Activity

**Figure 5:**
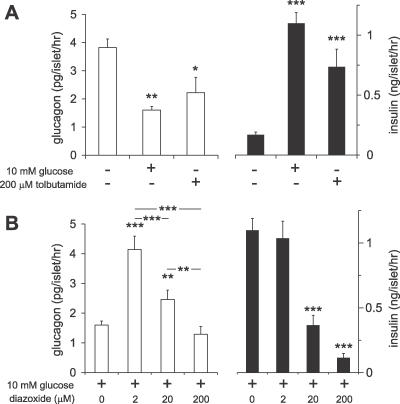
Glucagon Release from Isolated and Intact Human Islets Is Regulated by a K_ATP_ Channel-Dependent Pathway (A) Glucagon (open bars) and insulin (filled bars) release was measured from isolated human islets under control conditions and following addition of 10 mM glucose or 200 μM tolbutamide. (B) Glucagon (open bars) and insulin secretion (filled bars) measured in the presence of 10 mM glucose and increasing concentrations of diazoxide (0–200 μM). *, *p* < 0.05; **, *p* < 0.01; ***, *p* < 0.001, compared with controls, unless otherwise indicated.

Little work has been done to examine the mechanism of glucose-regulated glucagon secretion from isolated human islets [51]. Glucagon and insulin secretion from islets isolated from healthy donors was examined. Similar to above, glucagon secretion from human islets was suppressed by 58 ± 3% (*n* = 9, *p* < 0.05) upon raising the glucose concentration from 0 to 10 mM. Tolbutamide (200 μM) inhibited glucagon release by 43 ± 14% (*n* = 7, *p* < 0.05). At the same time, insulin secretion was increased 6.4- and 4.4-fold by glucose and tolbutamide stimulation, respectively (Figure 5A). Moderate re-activation of K_ATP_ channels with 2 μM diazoxide antagonized the glucose-induced suppression of glucagon release (*n* = 7, *p* < 0.01) (Figure 5B). A 10-fold higher concentration of diazoxide had significantly less stimulatory effect (*p* < 0.001 versus 2 μM diazoxide), and in the presence of 200 μM diazoxide, glucagon secretion was not different from that observed in the absence of the K_ATP_ channel activator. Importantly, the lowest concentration of diazoxide (2 μM), which produced the strongest stimulation of glucagon secretion, had no inhibitory effect on glucose-stimulated insulin secretion (*n* = 5), whereas 20 and 200 μM produced partial or complete inhibition of insulin secretion. The GABA_A_ receptor antagonist SR95531 had no effect on glucose-induced inhibition of glucagon secretion from human islets, and whole-cell voltage-clamp measurements indicate that human α cells, unlike human β and δ cells, express few if any GABA_A_ receptor Cl^−^ channels (M. Braun, R. Ramracheya, and P. Rorsman, unpublished data).

**Figure 6:**
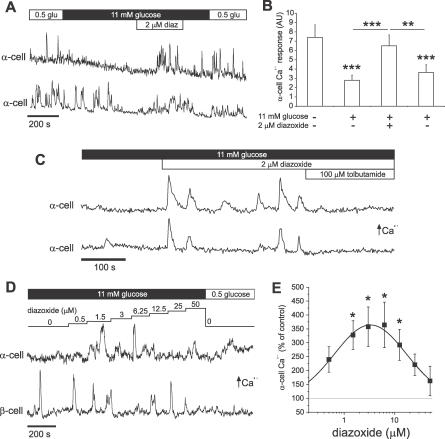
Intracellular Ca^2+^ Responses of α Cells within Intact Human Islets Are Regulated by a K_ATP_ Channel-Dependent Mechanism (A) Ca^2+^ responses measured in two human α cells within the same islet at 0.5 mM and 11 mM glucose (glu), in the presence of 2 μM diazoxide (diaz). (B) Summary of the Ca^2+^ responses at 0.5 mM glucose, at 11 mM glucose, in the presence of glucose (11 mM) and diazoxide (2 μM), and following the removal of diazoxide, but in the continued presence of 11 mM glucose. (C) The re-activation of human α-cell Ca^2+^ responses by 2 μM diazoxide was reversed upon application of the K_ATP_ channel antagonist tolbutamide (100 μM). (D) The effects of increasing concentrations of diazoxide on the Ca^2+^ response of a human α cell and β cell within the same islet exposed to 11 mM glucose. At the end of the experiment, diazoxide was withdrawn and glucose lowered to 0.5 mM. (E) Dose-response curve for the effect of diazoxide on α-cell Ca^2+^ responses. The grey horizontal line indicates the response with 11 mM glucose alone. *, *p* < 0.05; **, *p* < 0.01; ***, *p* < 0.001, compared with controls, unless otherwise indicated.

Examination of the Ca^2+^ responses of single α and β cells within intact human islets demonstrated that glucose decreased [Ca^2+^]_i_ by 62 ± 3% (*n* = 42 cells in 7 islets, *p* < 0.001) in α cells, and that this effect could be completely reversed by 2 μM diazoxide (Figure 6A and 6B). Furthermore, the re-activation of α-cell [Ca^2+^]_i_ responses by diazoxide could be prevented by application of 100 μM tolbutamide, confirming the role of K_ATP_ channels (Figure 6C). An analysis of the dose–response relationship to diazoxide demonstrated a maximally effective concentration of 1.7 μM (Figure 6D and 6E; *n* = 38 cells in 9 islets), far below the concentration necessary to block β-cell responses (Figure 6D). Increases in diazoxide above 20 μM blocked the [Ca^2+^]_i_ responses of α cells and β cells in parallel, consistent with the effect on glucagon and insulin release.

### Na^+^ Channels Regulate Low-Glucose–Stimulated Glucagon Secretion

**Figure 7:**
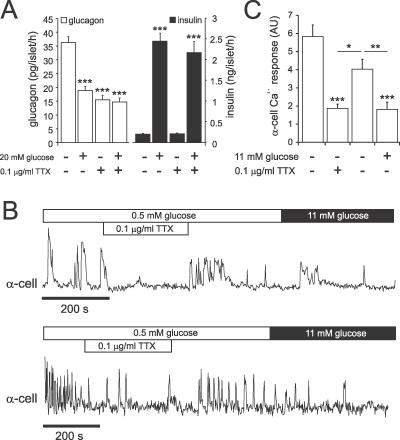
Glucagon Secretion and α-Cell Ca^2+^ Responses Are Dependent upon the Activity of Voltage-Dependent Na^+^ Channels (A) Glucagon (open bars) and insulin (filled bars) release from isolated mouse islets at 1 mM and 20 mM glucose, under control conditions and in the presence of the Na^+^ channel antagonist TTX (0.1 μg/ml). (B) Intracellular Ca^2+^ response of single α cells to 0.5 mM glucose. TTX (0.1 μg/ml) was included in the perfusion medium during the indicated period. (C) The effects of TTX and glucose on α-cell Ca^2+^ responses. Note that TTX inhibits Ca^2+^ responses as effectively as glucose and that the action is at least partially reversible. *, *p* < 0.05; **, *p* < 0.01; ***, *p* < 0.001, compared with controls, unless otherwise indicated.

The above data suggest that glucose-dependent inhibition of α-cell K_ATP_ channels is involved in the suppression of glucagon secretion. It is not clear however, how K_ATP_ channel inhibition and membrane depolarisation result in suppression of secretion. Unlike in β cells, α-cell Na^+^ channels are active in the physiological range of membrane potentials. Previous work from our group suggested that the voltage-dependent inactivation of Na^+^ channels, which in mouse α cells is half-maximal at −42 mV [43], contributes to cessation of electrical activity upon α-cell depolarisation [14]. We thus used the voltage-dependent Na^+^ channel antagonist tetrodotoxin (TTX) to test a role for these channels in α-cell function and glucagon secretion. TTX (0.1 μg/ml) suppressed glucagon release from mouse islets by 56 ± 5% (*n* = 10, *p* < 0.001) under low-glucose conditions (Figure 7A). This effect of TTX in the low-glucose condition was similar to what we observed with high-glucose stimulation alone. With TTX present, glucose was without further inhibitory action, suggesting that glucose inhibits glucagon secretion through a Na^+^ channel-dependent mechanism. TTX has no effect on glucose-stimulated insulin secretion in mouse islets (Figure 7A); again suggestive of a direct rather than indirect effect on α cells. In accordance with this, application of 0.1 μg/ml TTX to mouse islets reversibly abolished the α-cell [Ca^2+^]_i_ response evoked by low-glucose concentrations (Figure 7B and 7C; *n* = 18 cells in 4 islets), but had no effect on β-cell [Ca^2+^]_i_ (unpublished data).

### N-Type Ca^2+^ Channels Mediate the Ca^2+^ Influx That Triggers Glucagon Secretion and α-Cell Exocytosis

Downstream of Na^+^ channel activation, the opening of VDCCs allows Ca^2+^ into α cells, triggering the exocytosis of glucagon-containing vesicles. We applied whole-cell patch-clamp recordings to establish the α-cell Ca^2+^ channel complement. The integrated whole-cell Ca^2+^ current measured during 50-ms depolarisations from −70 mV to 0 mV amounted to 6.2 ± 0.8 pC (*n* = 15) under control conditions, 2.6 ± 0.8 pC (*n* = 10; *p* < 0.05) in the presence of 50 μM nifedipine, and 4.1 ± 0.5 pC (*n* = 12; *p* < 0.01) in the presence of 1 μM ω-conotoxin. Thus, L- and N-type Ca^2+^ channels account for 58% and 34% of the α-cell Ca^2+^ current, respectively.

**Figure 8:**
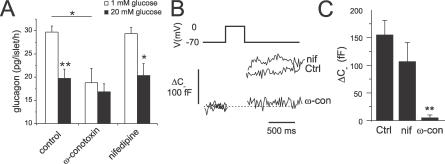
Glucagon Secretion Is Regulated by N-Type Ca^2+^ Channels (A) Glucagon release measured at 1 (open bars) and 20 mM glucose (filled bars) under control conditions and in the presence of 100 nM ω-conotoxin (middle) or 20 μM nifedipine (right). (B) Exocytosis was elicited by 500-ms depolarisations from −70 to 0 mV under control conditions (Ctrl) and in the presence of either nifedipine (50 μM; nif) or ω-conotoxin (1 μM; ω-con). (C) Summary of the exocytotic response under control conditions and in the presence of nifedipine and ω-conotoxin. *, *p* < 0.05; **, *p* < 0.01, compared with 1 mM glucose and the control capacitance response, unless indicated otherwise.

Figure 8A compares glucagon secretion at 1 and 20 mM glucose under control conditions and in the presence of 100 nM ω-conotoxin and 20 μM nifedipine, respectively. We found that whereas the L-type channel blocker nifedipine had no effect on glucagon release at 1 or 20 mM glucose, the N-type Ca^2+^ channel blocker ω-conotoxin inhibited the release of the hormone to a level similar to that of high glucose, tolbutamide, and TTX. Furthermore, glucose exerted no additional inhibitory action in the presence of ω-conotoxin. Thus, glucagon secretion stimulated by low-glucose levels depends principally on Ca^2+^ influx through N-type channels, although these only account for one third of the Ca^2+^ current. We confirmed this Ca^2+^ channel dependence by conducting high-resolution single-cell capacitance measurements of exocytosis (Figure 8B). Exocytosis elicited by 500-ms step depolarisations from −70 to 0 mV averaged approximately 150 fF (corresponding to the fusion of ∼75 secretory granules with the plasma membrane [43]) under control conditions (Ctrl). This response was not significantly affected by 50 μM nifedipine (nif), but was nearly abolished by 1 μM ω-conotoxin (ω-con; Figure 8C).

### Inactivation of N-Type Ca^2+^ Channels Underlie Glucose Inhibition of Glucagon Secretion

**Figure 9:**
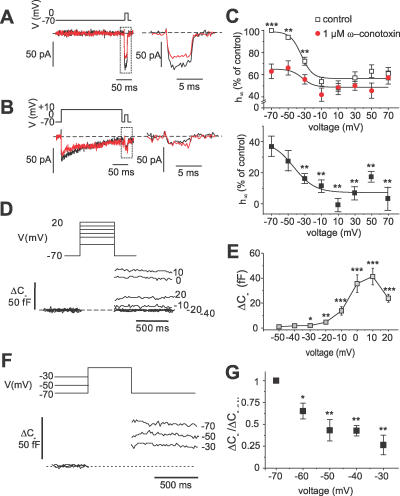
Voltage-Dependent Inactivation of α-Cell N-Type Ca^2+^ Currents and Exocytosis (A) N-type Ca^2+^ currents were evaluated during blockade of the L-type channels with isradipine (2 μM). The N-type channel antagonist ω-conotoxin (1 μM; red traces) reduced the Ca^2+^ current elicited by a step depolarisation from −70 to 0 mV (right). (B) As in (A), but the pulse to 0 mV was preceded by a 200-ms conditioning depolarization to +10 mV. ω-Conotoxin was without effect on the current measured during the depolarization to 0 mV under these conditions (right). (C) Top: peak Ca^2+^ currents measured in the presence of 2 μM isradipine alone (open squares) or together with 1 μM ω-conotoxin (red circles) during a depolarization to 0 mV following 200-ms conditioning pulses to between −70 and +70 mV. Lower: inactivation of the ω-conotoxin–sensitive component. Half-maximal inactivation of the N-type current was at −31 ± 6 mV (*n* = 5). (D) Exocytosis was elicited with 500-ms depolarisations from −70 mV to between −50 and 20 mV. (E) The voltage dependence of the exocytotic response. (F) Exocytosis elicited by 500-ms depolarisations to 0 mV from holding potentials of between −70 and −30 mV. (G) Summary of effects of holding potential on exocytotic responses elicited by depolarisations to 0 mV. Data have been normalized to responses obtained using a holding potential of −70 mV. *, *p* < 0.05; **, *p* < 0.01; ***, *p* < 0.001, compared with ω-conotoxin (C, top) or with the initial response.

We next examined the mechanism by which N-type channels regulate glucagon release in response to membrane depolarisation/hyperpolarisation in mouse α cells. Non–L-type (isradipine-resistant) Ca^2+^ currents were elicited by step depolarization to 0 mV in the absence (black) or presence (red) of 1 μM ω-conotoxin following a 200-ms conditioning pulse to −70 mV (Figure 9A) or +10 mV (Figure 9B). It is evident that the ω-conotoxin–sensitive component was abolished by the +10 mV conditioning pulse. Figure 9C summarizes the relationship between the conditioning voltage and the Ca^2+^ current amplitude in the absence (open squares) and presence (circles) of ω-conotoxin (upper panel). The ω-conotoxin–sensitive N-type Ca^2+^ current component is shown in the lower panel of Figure 9C, and underwent voltage-dependent inactivation that was half-maximal (V_0.5_) at −31 ± 6 mV (*n* = 6). A residual, ω-conotoxin–insensitive Ca^2+^ current also appears to undergo voltage-dependent inactivation (Figure 9C). This accounts for less than 15% of the inactivating current, and may be attributable to T-type Ca^2+^ channels that have been reported in α cells [14].

In Figure 9D, we examined the voltage-dependent *activation* of α-cell exocytosis using step-wise depolarisations (500 ms) from −70 mV to between −50 and 20 mV (Figure 9D). The capacitance response versus voltage relationship (Figure 9E) demonstrates a marked increase in the capacitance response between −10 and 10 mV. Thus any reduction in action potential amplitude within this range would severely attenuate α-cell exocytosis.

The voltage-dependent *inactivation* of α-cell exocytosis was examined next (Figure 9F). Cells were held at conditioning potentials between −70 and −30 mV, after which exocytosis was elicited by depolarisation to 0 mV. Exocytosis stimulated from the −30 mV conditioning potential was only 25% of that elicited from −70 mV (Figure 9G). This voltage-dependent decline in exocytosis is attributable to the voltage-dependent inactivation of the N-type Ca^2+^ channels. The fact that inhibition of exocytosis appears to occur at more-negative voltages than that documented for the inactivation of the N-type Ca^2+^ channels can be attributed to the brevity of the conditioning pulses in Figure 9A–9C (200 ms), whereas in Figure 9G the holding potential was varied.

## Discussion

Regulated glucagon secretion from pancreatic α cells is a major component of the counter-regulatory response to hypoglycaemia. Diabetes mellitus is associated with defects of glucagon secretion that exacerbate the consequences of impaired insulin secretion [2–4]. The mechanism regulating the release of this important hormone is incompletely understood and currently the source of much debate. In the present study we examined glucagon secretion and the [Ca^2+^]_i_ response of in situ α cells of isolated rodent and human islets. We further characterised the Ca^2+^ currents and capacitance changes of single isolated α cells, to dissect the mechanism regulating downstream control of glucagon release.

Previous work with genetic mouse models, including SUR1^−/−^ [13,34,35] and Kir6.2^−/−^ [16] mice, suggests an important role for K_ATP_ channels in glucagon secretion. Three pieces of evidence argue for the importance of *islet* K_ATP_ channels in glucagon secretion. First, introduction of the Tyr12STOP mutation into the K_ATP_ channel subunits in the Kir6.2Y12X islets results in suppression of glucagon secretion at low-glucose levels and causes loss of glucose-induced inhibition of secretion. Second, the K_ATP_ channel inhibitor tolbutamide when applied at maximally effective concentrations inhibits glucagon secretion from isolated islets. Third, a low dose of the K_ATP_ channel activator diazoxide restores α-cell Ca^2+^ responses and glucagon secretion in high-glucose conditions. What is not immediately clear from these arguments, and from the previous SUR1^−/−^ genetic studies, is whether glucagon is being controlled by the α-cell or the β-cell K_ATP_ channels, as the latter may regulate α-cell function indirectly via paracrine pathways. We therefore examined insulin and glucagon secretion, and the Ca^2+^ responses of α and β cells in situ in parallel to determine the functional relationship between these cells. Furthermore, we measured the glucagon response to glucose during blockade of putative paracrine signalling pathways.

Studies on rat islets support an important role for paracrine signals as regulators of glucagon release [33,52,53], whereas the case for paracrine regulation of glucagon secretion from mouse and human α cells is less clear [9,10,14,17–21,27]. Although it is clear that Zn^2+^, GABA, and somatostatin can exert a paracrine control of glucagon secretion under certain conditions, the data shown here firmly establish that glucose can suppress glucagon secretion independently of these pathways as demonstrated in Figure 1 (and in [47]). Furthermore, and in agreement with recently published results [47], maximal inhibition of glucagon release occurs at levels equal to or lower than 5 mM glucose, whereas the stimulation of insulin release requires glucose levels greater than 5 mM (Figure 3), suggesting that products of β-cell secretion are not required for suppression of glucagon release.

This conclusion is further underpinned by the significant discordance between insulin and glucagon release, and the β- and α-cell Ca^2+^ responses, under several conditions: (1) low doses of the K_ATP_ channel opener diazoxide that stimulate glucagon secretion while not affecting insulin release; (2) high doses of diazoxide at which suppression of β-cell secretion would be expected to elevate glucagon release; (3) in the Kir6.2Y12X islets; and (4) in response to the Na^+^ channel blocker TTX. Therefore, our data argue that glucagon-producing α cells possess an intrinsic mechanism for regulation by glucose and that involves K_ATP_ channels. This is at variance with the data of Liu et al. [12] who report that tolbutamide has no effect on [Ca^2+^]_i_ in single, isolated α cells, but in agreement with the conclusion of Ostenson et al. [54], that sulfonylureas can inhibit glucagon secretion by a direct, non-paracrine mechanism.

**Figure 10:**
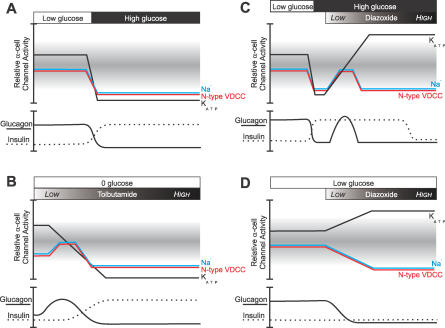
A Model for the Suppression of Glucagon Secretion by an Intrinsic α-Cell Pathway Schematic representation of the effects of glucose, tolbutamide, and diazoxide on α-cell K_ATP_, Na^+^, and N-type Ca^2+^ (VDCC) channel activities and glucagon secretion is shown. The insulin response is also shown for comparison with our experimental results (dashed lines, lower panels). The grey gradient represents a “window” of α-cell K_ATP_ channel activity that supports the activation of Ca^2+^ and Na^+^ channels. Above this window, the cell is hyperpolarized and Ca^2+^ and Na^+^ channel activation is prevented, whereas K_ATP_ channel activity below this window depolarizes the cell and causes voltage-dependent inactivation of Ca^2+^ and Na^+^ channels. (A) High-glucose concentration reduces α-cell K_ATP_ channel activity, reducing glucagon secretion. (B) Graded application of tolbutamide (in zero glucose) transiently increases glucagon secretion as K_ATP_ channel activity is reduced through, and eventually below, the window supporting glucagon release. (C) The graded application of diazoxide in high-glucose conditions increases α-cell K_ATP_ channel activity into, and then above the window supporting glucagon secretion. The result is a transient “re-activation” of glucagon secretion at low-diazoxide concentrations. (D) In low-glucose (1–2 mM) conditions, graded application of diazoxide increases K_ATP_ channel activity above the window supporting glucagon secretion, causing a monotonic inhibition of glucagon release.

On the basis of our findings, we propose that α-cell glucagon secretion occurs within a narrow window of intermediate K_ATP_ channel activity (and thus membrane potential) (Figure 10). That is, if the α cell is either too hyperpolarised (maximal K_ATP_ activity) or too depolarised (maximal K_ATP_ inhibition), then glucagon secretion is suppressed. This is supported by the biphasic effects of both diazoxide and tolbutamide. Whereas the former (in high glucose) brings the α cell in a dose-dependent manner through the membrane potential window supporting glucagon secretion in the depolarised (low K_ATP_) to hyperpolarised (high K_ATP_) direction (Figure 10C), the latter (in zero glucose) brings the α cell through the window in the opposite direction (from hyperpolarised to depolarised) (Figure 10B). Thus, glucose likely leads to the suppression of glucagon secretion by depolarising the α-cell membrane potential above the range that supports glucagon secretion (Figure 10A). Indeed, in some (but not all, see [55]) studies, glucose was found to depolarize mouse and rat α cells and reduce action potential amplitudes [13,52]. It is interesting that whereas low concentrations of glucose were without stimulatory effect, tolbutamide (0.1–1 μM) stimulated glucagon release beyond that observed at zero glucose. Thus, *small* depolarisations (as previously documented for arginine [13]) exert a positive “chronotropic” effect in α cells and thus stimulate glucagon release. The fact that glucose did not share this ability indicates that the depolarisation produced by 1 mM glucose results in sufficient inactivation of the currents to balance any increase in action potential frequency.

Previous human studies have employed pharmacological K_ATP_ channel antagonists [23,24] or agonists [25,26] to examine the regulation of glucagon release in vivo. These were interpreted with the assumption that pharmacological K_ATP_ modulation only affects the β cells, and that any change in glucagon release was therefore secondary to altered β-cell function. Our data establish that K_ATP_ channel modulation has dramatic and direct effects on glucagon secretion and Ca^2+^ signalling in human α cells under conditions in which insulin secretion is unaffected. Thus, the in vivo manipulation of α-cell K_ATP_ channel activity in the above studies may well have involved direct effects on α-cell function that contributed to the observed changes in glucagon release.

The importance of the human K_ATP_ channel pathway for glucagon release is nicely highlighted by a study investigating the effects of the common Glu23Lys (E23K) polymorphism of the Kir6.2 subunit of the K_ATP_ channel on insulin and glucagon secretion in non-diabetic human patients [29]. This variant of the channel leads to a slight decrease in the ATP sensitivity of the channel. The functional significance of this was examined by comparing hormone release during hyperglycaemic clamps in individuals carrying the polymorphism or not. Although insulin secretion in homozygous Glu23Lys individuals was not different from controls, glucose-induced suppression of glucagon release was blunted [29]. This becomes understandable in light of the effect of diazoxide on isolated human islets (Figures 4 and 5). Half-maximal activation of K_ATP_ channels occurs at diazoxide concentrations of 20–100 μM (depending on the intracellular ATP level) [56]. If the Glu23Lys polymorphism increases K_ATP_ channel activity to the same extent as 0.3–1.5 μM diazoxide, the concentration at which an effect on glucagon release is first seen, then the effect will be very difficult to detect with electrophysiology, perhaps explaining why some studies have failed to detect a functional effect of the polymorphism (reviewed in [57]). Nevertheless, such small changes can have significant biological effects, as illustrated by the glucagon release data, and may contribute to pathological states such as impaired glucose tolerance and diabetes. Thus, the reduced ability of glucose to inhibit glucagon secretion in individuals carrying the Glu23Lys variant of the K_ATP_ channel likely results from the failure of these channels to undergo complete inhibition in response to glucose.

We have proposed that glucagon secretion is stimulated within a window of intermediate α-cell K_ATP_ channel activity, and that glucagon release is suppressed by either increases or decreases in K_ATP_ channel activity. How is this accomplished? Briefly, we suggest that this window is the result of (1) the ability of intermediate K_ATP_ channel activity to support regenerative electrical responses through the activation of voltage-dependent Na^+^ and N-type Ca^2+^ channels (grey in Figure 10); (2) the failure of the Na^+^ and Ca^2+^ channels to activate when α cells are hyperpolarised by the opening of a major fraction of K_ATP_ channels (above the grey in Figure 10); and (3) the voltage-dependent *inactivation* of the Na^+^ [14] and N-type Ca^2+^ channels when K_ATP_ channels are closed and the α-cell membrane potential is depolarised (below the grey in Figure 10). Thus, the differential responsiveness of α and β cells to diazoxide does not result from differential sensitivities of the K_ATP_ channels in these cells, but to the downstream responses to titrated K_ATP_ channel activity.

One important difference between mouse α and β cells is that whereas the latter rely exclusively on voltage-gated Ca^2+^ channels for the upstroke of the action potentials, glucagon-producing α cells are equipped with voltage-gated Na^+^ channels. These channels undergo voltage-dependent inactivation at voltages more positive than −50 mV [14]. This will reduce the action potential amplitude, and indeed it is reported that glucose reduces the peak voltage in α cells from +11 mV to −1 mV [13]. This will in itself result in an approximately 35% reduction of exocytosis, which is steeply dependent on voltage between −10 and +10 mV (Figure 9E). The functional significance of this is illustrated by the observations that glucagon secretion and α-cell Ca^2+^ responses at low-glucose concentrations are suppressed by the Na^+^ channel blocker TTX. The exact ion channel complement of human α cells remains to be established. However, the fact that glucagon secretion from human islets shows the same bell-shaped diazoxide concentration dependence as in mouse islets suggests that the depolarization-induced inactivation of ion channels involved in α-cell regenerative electrical activity underlies glucose-induced suppression of glucagon secretion in man as well.

Although L-type VDCCs mediate the majority of Ca^2+^ current in α cells, this work and previous studies [33,44,45] demonstrate that it is the N-type (ω-conotoxin–sensitive) VDCCs that mediate the Ca^2+^ influx necessary for α-cell exocytosis and glucagon secretion under hypoglycaemic conditions. We now show that the α-cell N-type VDCCs are also subject to voltage-dependent inactivation at voltages more positive than −50 mV and furthermore that this is associated with reduced exocytotic capacity. It is pertinent that N-type VDCC-deficient mice exhibit reduced serum glucagon levels and improved glucose tolerance despite a parallel reduction in plasma insulin [44]. Although Ca^2+^ influx through L-type Ca^2+^ channels does not appear to contribute much to glucagon secretion under the experimental conditions used in this study, these channels become the predominant conduit of Ca^2+^ entry in the presence of agents increasing cAMP [41] (unpublished data). The mechanism underlying this switch in Ca^2+^ channel dependence remains obscure, but may depend on the strength of depolarisation because glucagon secretion stimulated by strong depolarisation with increased K^+^ in combination with K_ATP_ channel block can be prevented by inhibition of L-type Ca^2+^ channels [47].

We finally point out that the model we propose here for the control of glucagon secretion shares many features with what is known about the metabolic control of insulin secretion. This in turn means that processes that interfere with the ability of, for example, glucose to stimulate insulin secretion will have the opposite effect on glucagon secretion. This would provide a simple explanation for the fact that both insulin and glucagon secretion become perturbed in diabetes and why oversecretion of glucagon exacerbates the hyperglycaemic effects of insufficient insulin secretion.

## Materials and Methods

### Islet isolation and culture.

Islets from female NMRI mice were isolated by collagenase digestion and cultured in RPMI-1640 media (5 mM glucose) at 37 °C and 5% CO_2_ for 2−24 h prior to secretion or intracellular Ca^2+^ assays. For single-cell studies, islets were dispersed in a Ca^2+^-free buffer and plated in 35-mm plastic dishes. Generation of the Kir6.2Y12X mice, which possess a Tyr12STOP mutation in the *Kcnj11* gene on a BALB/c background, has been described previously [48]. This results in nonfunctional K_ATP_ channels in both α and β cells. These animals are not overtly diabetic, exhibiting normal fasting glucose, but are glucose intolerant (R. Cox, unpublished data). For these experiments, age- and weight-matched wild-type C3HB mice were used as controls because the Kir6.2Y12X mice were back-crossed into this background over several generations. Human islets from four healthy donors were obtained from the Oxford DRWF Human Islet Isolation Facility. Experiments were performed in duplicate or triplicate using islets from each donor. Human islets were cultured in RPMI-1640 (10 mM glucose) at 37 °C and 5% CO_2_, and experiments were performed within 48 h of isolation.

### Intracellular Ca^2+^ measurements.

Intact islets were loaded with fluo-4-AM (1 μM) and fura red (5 μM) in 0.5 mM glucose buffer (see below) with 0.01% pluronic acid for 30 min at 37 °C. Islets were fixed with a wide-bore holding pipette within a continuously superfused and temperature-controlled (37 °C) bath on an Axioskop 2 FS-mot microscope (Carl Zeiss, http://www.zeiss.com). The perfusion buffer contained (in mM): 140 NaCl; 3.6 KCl; 2 NaHCO_3_; 0.5 NaH_2_PO_4_; 0.5 MgSO_4_; 5 HEPES; 2.5 CaCl_2_; 0.5, 3, or 11 glucose; (pH 7.4 with NaOH). Laser scanning confocal microscopy was performed using an LSM 510meta system (Zeiss). Excitation was with a 488-nm argon laser, and emitted fluorescence was collected through 500–550-nm and 650–710-nm band-pass filters for the fluo-4 and fura red (FuraR) signals, respectively. Increases in intracellular Ca^2+^ are displayed as upward deflections. Images were acquired at 1.5-s intervals. Individual cells were selected as regions of interest, and the average ratio intensity (RI = [Fluo+1]/[FuraR+20] × 128+1) of these were analysed over time with Origin v7.0220 (OriginLab Corporation, http://www.originlab.com). Prior to experimental recordings, islets were perfused for 10 min with the appropriate control solution, and Ca^2+^ responses were monitored periodically during this time to ensure that responses (or lack thereof) were stable prior to beginning the experimental recording. Ca^2+^ responses were determined by baseline subtraction and calculation of the integrated response (i.e., the area under the curve). The slope of this response, reported here as arbitrary units (AU), was calculated for the final 60%–90% of a given treatment period to allow for equilibration of the responses.

### Hormone release.

Insulin and glucagon secretion were measured as described elsewhere [45]. Briefly, batches of ten freshly isolated islets were pre-incubated in 1 ml of Krebs–Ringer buffer (KRB) supplemented with 1 mM glucose for 30 min (rodent) or 1 h (human) followed by 1-h incubation in 1 ml of test KRB medium supplemented as indicated. For experiments in which Zn^2+^ was chelated or GABA_A_ receptors were antagonised (Figure 1), the Ca^2+^-EDTA and SR-95531, respectively, were present in both the pre-incubation and test solutions. When extracellular KCl was increased, NaCl was correspondingly reduced to maintain iso-osmolarity.

### Single-cell capacitance and ion current measurements.

Whole-cell currents and exocytosis were recorded using an EPC-9 patch-clamp amplifier (HEKA Electronics, http://www.heka.com) and Pulse software (version 8.50). Single α cells were identified by their small size and Na^+^ current inactivation properties [58]. This method was validated by combining the electrophysiological recordings with subsequent immunostaining for glucagon and insulin. The extracellular medium contained (in mM): 118 NaCl; 20 TEA-Cl (tetraethylammonium chloride); 5.6 KCl; 2.6 CaCl_2_; 1.2 MgCl_2_; 5 HEPES (pH 7.4 with NaOH); and 5 glucose. Except for Figure 8B and 8C, in which the standard whole-cell configuration was used, the electrophysiological measurements were conducted using the perforated patch technique, and the pipette solution contained (in mM): 76 Cs_2_SO_4_; 10 NaCl; 10 KCl; 1 MgCl_2_; and 5 HEPES (pH 7.35 with CsOH). Exocytosis was monitored as changes in α-cell capacitance using the software-based lock-in function of the Pulse software. The standard whole-cell measurements (Figure 8B and 8C) were conducted using a pipette solution (dialyzing the cell interior) consisted of (in mM) 125 CsOH; 125 glutamate; 10 CsCl; 10 NaCl; 1 MgCl_2_; 5 HEPES (pH 7.15 with KOH); 3 Mg-ATP; and 25 μmol/l EGTA (measured resting free Ca^2+^, ∼0.2 μmol/l). Pulses were applied at low frequency (<0.05 Hz) to allow the exocytotic capacity to recover fully between the pulses.

### Statistical analysis.

Data are presented as means and standard errors. Significance was examined by either the unpaired *t*-test or by multiple-comparisons analysis of variance (ANOVA) and post-test, as appropriate.

## Abbreviations

[Ca^2+^]_i_: intracellular Ca^2+^
FuraR: fura red
GABA: γ-aminobutyric acid
K_ATP_: ATP-dependent K^+^, TTX, tetrodotoxin
VDCC: voltage-dependent Ca^2+^ channel
